# The efficacy of traditional Chinese medicine sequential therapy in non-alcoholic fatty liver disease

**DOI:** 10.12669/pjms.41.2.11352

**Published:** 2025-02

**Authors:** Li Zhang, Yuanfang Qian, Hongdi Wu, Hongyan Xu

**Affiliations:** 1 Li Zhang Department of Infectious Diseases, Tongde Hospital of Zhejiang Province, Hangzhou, Zhejiang Province 310012, P.R. China; 2 Yuanfang Qian Department of Nursing, Tongde Hospital of Zhejiang Province, Hangzhou, Zhejiang Province 310012, P.R. China; 3 Hongdi Wu Department of Infectious Diseases, Tongde Hospital of Zhejiang Province, Hangzhou, Zhejiang Province 310012, P.R. China; 4 Hongyan Xu Department of Nursing, Tongde Hospital of Zhejiang Province, Hangzhou, Zhejiang Province 310012, P.R. China

**Keywords:** Non-alcoholic fatty liver disease, Sequential therapy, Traditional Chinese Medicine

## Abstract

**Objective:**

Sequential therapy in traditional Chinese medicine (TCM) refers to a combination of internal and external treatment methods in a certain order based on syndrome differentiation and therapy. The aim of this study was to evaluate the efficacy of TCM sequential therapy that is given on the basis of conventional management in patients with non-alcoholic fatty liver disease (NAFLD).

**Methods:**

Medical records of one hundred NAFLD patients who received treatment at Zhejiang Provincial Tongde Hospital between February 2023 and April 2024 were retrospectively analyzed. Of them, 48 patients received routine intervention (Control group), and 52 patients were additionally treated by sequential TCM therapy (TCM group). The levels of blood lipid indicators, liver function indicators, TCM syndrome efficacy, and quality of life (QOL) scores were compared between the two groups before and after the intervention.

**Results:**

After the intervention, the levels of blood lipid and liver function indicators in both groups were significantly improved compared to preintervention and were considerably better in the TCM compared to the Control group (*P*<0.05). The overall efficacy was higher in the TCM group (94.23%) compared to the Control group (79.17%) (*P*<0.05). After the intervention, the QOL scores of both groups increased and were significantly higher in the TCM group than in the Control group (*P*<0.05).

**Conclusions:**

For NAFLD patients, adopting TCM sequential therapy in addition to the routine disease management strategies can effectively regulate patients’ blood lipid levels, restore liver function, improve disease treatment effectiveness, and improve the quality of life.

## INTRODUCTION

Non-alcoholic fatty liver disease (NAFLD) is mainly characterized by diffuse hepatic steatosis caused by factors other than alcohol.^1^,^2^ In recent years, with the change in diet, standards of living, and social environment, the incidence of NAFLD is on the rise, and the patient population is becoming increasingly younger.^3^ While standard clinical management of NAFLD patients, such as dietary and exercise guidance, has achieved certain results,^4^,^5^ it lacks personalization. Furthermore, precise interventions are often difficult to implement due to the dynamic changes in the patient’s condition.^4^–^6^

Traditional Chinese Medicine (TCM) is commonly used in the treatment of hepatic diseases in Asia.^7^ In TCM, the treatment of NAFLD mainly focused on the holism of hepatoprotection and differentiates the symptoms of NAFLD as the following five types: stagnation of liver-qi, congestion of turbidity, accumulated damp-heat, spleen-deficiency and phlegm-turbid stagnation, and deficiency of liver and kidney.^8^ TCM accentuates dialectical intervention and proposes sequential therapy based on dynamic differentiation and treatment at different disease stages.^9^ TCM sequential therapy refers to a combination of internal and external treatment methods in a certain order based on syndrome differentiation and therapy.^9^,^10^ It emphasizes timely adjustment of intervention plans based on changes in the patient’s condition and implements comprehensive and systematic interventions in a certain order and steps. This regulates physical fitness and consolidates the effectiveness of disease intervention.^9^,^10^ TCM sequential therapy has been successfully used in treating various diseases. Zhang XL et al.^11^ used TCM sequential therapy for infertile patients with repeated implantation failures and showed that the implantation rate, biochemical pregnancy rate, and clinical pregnancy rate were higher in the TCM group compared to the control group of patients who received conventional intervention. In addition, Pan G et al.^12^ explored the intervention value of TCM sequential therapy in patients with acute myocardial infarction after percutaneous coronary intervention (PCI) and demonstrated that TCM sequential therapy was beneficial for improving heart function and regulating heart rate, and played an important role in alleviating clinical symptoms and improving quality of life.

However, while some studies assessed the efficacy of TCM in treating patients with NAFLD,^13^-^15^ the application value of TCM sequential therapy in NAFLD has not been widely confirmed. This study intends to clarify the intervention value of combining TCM sequential therapy with routine management methods in patients with NAFLD.

## METHODS

This retrospective cohort study included clinical records of NAFLD patients at Zhejiang Provincial Tongde Hospital from February 2023 to April 2024. Patients received the routine intervention (Control group) or sequential TCM therapy in addition to routine intervention (TCM group).

### Ethical approval:

This study was approved by the Ethics Committee of Zhejiang Tongde Hospital (No. 2024-084K, Date: June 26, 2024) and exempted patients from the need for informed consent.

### Inclusion criteria:

- Patients met the diagnostic criteria for NAFLD.^1^
- Patients aged 18 to 65 years.
- Patients with complete clinical data.

### Exclusion criteria:

- Patients with ascites.
- Patients with cirrhosis.
- Breastfeeding and pregnant women.
- Patients with respiratory, endocrine, and cardiovascular diseases.
- Allergic constitution.
- Patients with mental system disorders.

### Treatment methods:

The control group received routine intervention alone, and the TCM group received sequential TCM therapy plus based on routine intervention. Both groups were given a standardized education on diet and exercise, followed by a follow-up examination once every two weeks, and the duration of the intervention was 12 weeks. The protocols for the routine intervention and sequential TCM therapy were as follows.

### Routine intervention:

- Lifestyle guidance, including correcting unhealthy lifestyles and behaviors, correcting sleep disorders, excessive sitting and lack of movement, and picky eating. Patients were informed of the - - Dietary guidance. Patients were guided to maintain a balanced diet. The recommended energy distribution was as follows: 25% fat, 60-65% carbohydrates, and 15-20% protein. Patients were instructed to consume meat<50g, dairy products<250ml, and one egg. Alcohol and fatty meats were prohibited. Daily calorie requirement was calculated based on the patient’s condition (controlled at 121 kJ/kg). For individuals with a body mass index (BMI) ≥ 24 kg/m2, the daily calorie intake was reduced by 500-1000 kcal.
- Sports guidance. The patient’s personal preferences and interests, weight, and physical condition were considered, and personalized exercise plans were developed, including playing ball, going up and down stairs, medium to fast walking, skipping rope, jogging, swimming, dancing, and cycling. The duration of a single continuous aerobic exercise was between 45-60 minutes. Patients were instructed to gradually increase walking exercises from 5000 steps per day to 7000-8000 steps per day.

### TCM Sequential Therapy:

Physicians and nurses received training on TCM sequential therapy. Upon admission, patients underwent TCM syndrome differentiation with responsible nurses and physicians to clarify specific syndrome types. Corresponding intervention measures such as herbal, martial arts, acupressure, and appropriate TCM techniques for health preservation were summarized in detail in Table-I.

**Table-I:** Guidelines for Sequential Treatment in TCM.

Symptoms of TCM	Herbal (Note: use water to decoct twice, filtered liquid after togetder, simmer to 200mL; Twice a day, 100mL each time)	Martial arts (Note: 30 minutes/time, 2 times/day)	Acupressure (Note: 5 times/week, 30 minutes/time)	Appropriate techniques in TCM
Stagnation of liver-qi	Angelica 9~12g, White Paeony Root 9~12g, Liquorice 3~6g, Tangerine peel 3~6g,Cassia seed 12~15g, hawthorn 3~6g, Codonopsis 6~9g, Salvia 3~6g, Atractylodes 3~6g, tuckahoe 3~6g	Baduanjin, Yijinjing	Taichong (LR03), qimeng (LA14), Xuehai (SP10), hangjian (LR2), Ganshu (BL18)	Massage, balance cupping, meridian tapping ( 10 minutes/time, 2 times/day)
Congestion of turbidity	Pinellia tuber 9~12g, grifola 12~15g, lentil 9~12g, Tangerine peel 3~6g, tuckahoe 12~15g, yam 3~6g, Corn whiskers 3~6g, Coix lacryma 3~6;	Baduanjin, Tai Chi	Gongsun (SP4), taixi (KI3), yinlingquan (SP9), fenglong (ST40)	Acupoint application, ear point application, and TCM packaging (30 minutes/time, once a day)
Accumulated damp-heat	Corn whiskers 9~12g, lentil 3~6g, tuckahoe 6~9g, Coix lacryma12~15g, Lotus leaf 6~9g, bupleurum 12~15g, licorice 3~6g	Baduanjin, Tai Chi	fenglong (ST40), neiting (ST44), qihai (RN6), Zhongwan (RN12)	Scraping therapy, acupoint application, bloodletting therapy (15 minutes/time, once a day)
Spleen-deficiency and phlegm-turbid stagnation	Angelica 9~12g, salvia 12~15g, walnuts 9~12g, safflower 12~15g, Panax notoginseng 6~9g, motherwort 3~6g	Wuqinxi, Baduanjin, Tai Chi	Sanyinjiao (SP06), diji (SP8), Xuehai (SP10), Hegu (LI04)	Balanced cupping therapy, soaking therapy, thermosensitive moxibustion (15 minutes/time, 1 time/day)
Deficiency of liver and kidney	Medlar 12~15g, salvia 12~15g, Polygonatum sibiricum Red 12~15g, tuckahoe 9~12g, Cuscuta chinensis Lam 3~6g, Fructus Ligustri Lucidi 12~15g, Taxillus chinensis 3~6g, desertliving cistanche herb 3~6g, Twotooth Achyranthes Root12~15g	Tai Chi	Sanyinjiao (SP06), taixi (KI3), Taichong (LR03), zhusanli (ST36)	Acupoint application, ear acupressure, moxibustion (30 minutes/time, once a day)

### Collected information:

- Baseline date including gender, age, BMI, disease duration, severity of illness and education level.
- Blood lipid index levels. Approximate 2ml of the patient’s blood and BS-390 fully automatic biochemical analyzer (brand: Mindray; Shenzhen, China) were used to measure the levels of low-density lipoprotein cholesterol (LDL-C), triglycerides (TG), and total cholesterol (TC).
- Level of liver function indicators. Briefly, 2ml of serum from fasting venous blood was used to measure levels of Gamma-Glutamyl Transferase (γ-GT), alanine aminotransferase (ALT), and aspartate aminotransferase (AST) by enzyme-linked immunosorbent assay. The reagent kit was purchased from Shanghai Biyun Tian Biotechnology Co., Ltd.

### Intervention effect:

The Consensus Opinion on TCM Diagnosis and Treatment of NAFLD (2017)^16^ was used for reference: Significant effect - Blood lipids return to normal, signs and clinical symptoms basically disappear, and TCM syndrome scores decrease by more than 95%; Effective: Significant improvement in blood lipids levels, physical signs, and clinical symptoms, with a 70% to 95% reduction in TCM syndrome scores; Invalid: Blood lipids, physical signs, and clinical symptoms have improved, and the TCM syndrome score has decreased by 30% to 70%. Overall effective rate = [(number of significantly effective cases + number of effective cases)/total cases] ×100%.

Quality of life rating, estimated using the Quality of Life Scale (QOLS), with a total of 100 points. Higher scores indicate a better quality of life.^17^

### Statistical Analysis:

All data analyses were conducted using SPSS25.0 software (IBM Corp, Armonk, NY, USA) and PRISM8.0 software (GraphPad, San Diego, USA). The Shapiro Wilk test was used to evaluate the normality of the evaluation data. Normal distribution data were reported as mean ± standard deviation, with paired t-test for intra-group comparison before and after treatment and independent sample t-test for inter-group comparison. Non-normally distributed data were reported as median and interquartile ranges, with Mann-Whitney U test for inter-group comparison, and Wilcoxon test for intra-group comparison. Categorical variables were reported as frequency and percentage, with Chi-square test for comparison between the two groups. *P*<0.05 indicated statistically significant difference.

## RESULTS

A total of one hundred NAFLD patients met the criteria for this study. Among them, there were 63 males and 37 females, with an age range of 28-64 years and a median age of 50.5 years (44-55). The Control group included 48 cases; the TCM group included 52 cases with no significant difference in baseline data between the two groups (*P*>0.05) (Table-II).

**Table-II:** Comparison of baseline data between two groups.

Baseline data	TCM group (n=52)	Control group (n=48)	χ^2^/t/Z	P
Male (yes), n(%)	31 (59.6)	32 (66.7)	0.532	0.466
Age (years), M(P25/P75)	51 (46.5-55)	50 (42-55.5)	-0.656	0.512
BMI (kg/m^2^), Mean ± SD	23.15±3.08	22.70±3.37	0.706	0.482
Disease duration (years), Mean ± SD	3.84±1.29	4.11±1.32	-1.037	0.302
***Degree of illness, n (%)***				
Light	21 (40.4)	15 (31.3)	1.819	0.403
Moderate	24 (46.1)	22 (45.8)		
Severe	7 (13.5)	11 (22.9)		
***Educational level, n (%)***				
Primary school	19 (36.5)	13 (27.1)	1.133	0.567
Middle school and high school	22 (42.3)	22 (45.8)		
College degree or above	11 (21.2)	13 (27.1)		

**Fig.1:**
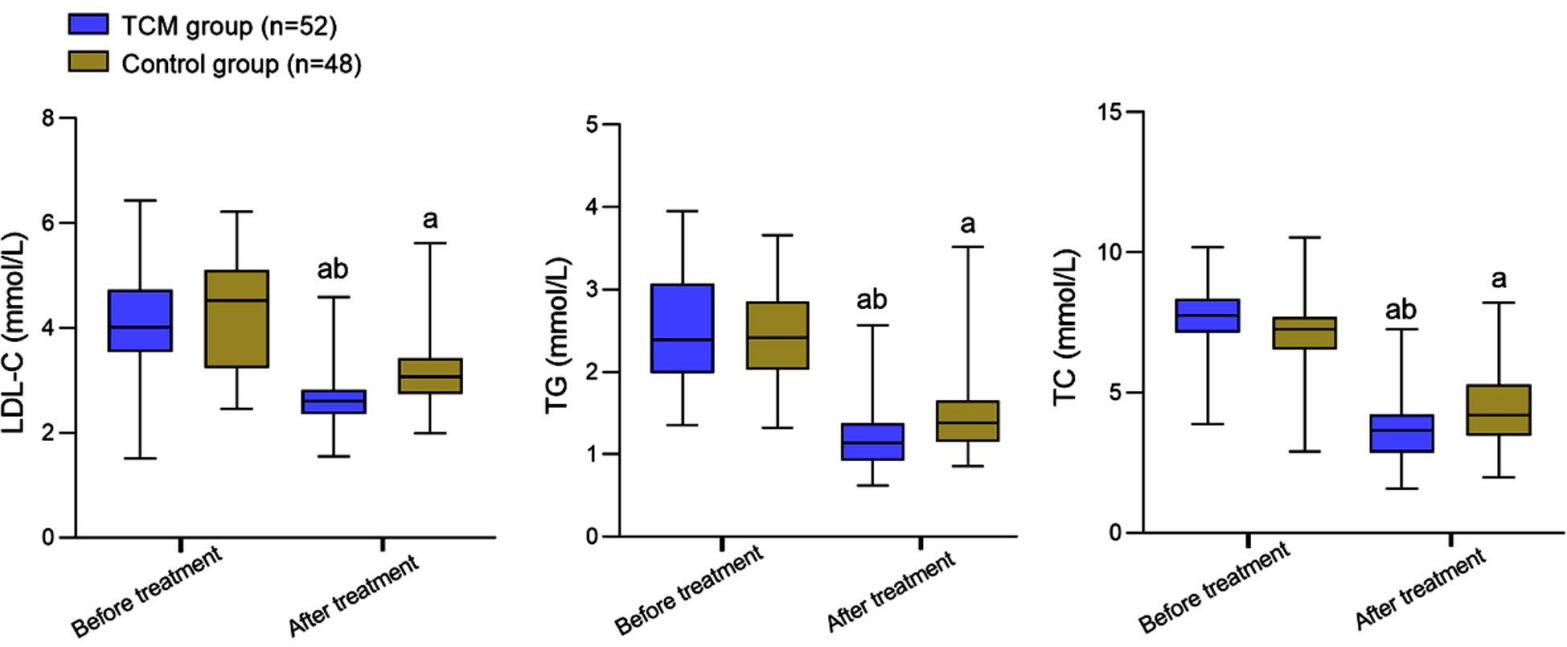
Comparison of blood lipid levels between two groups; Compared with before treatment, ^a^*P*<0.05; Compared with the Control group, ^b^*P*<0.05; traditional Chinese medicine (TCM); total cholesterol (TC); low-density lipoprotein cholesterol (LDL-C); triglyceride (TG).

**Fig.2:**
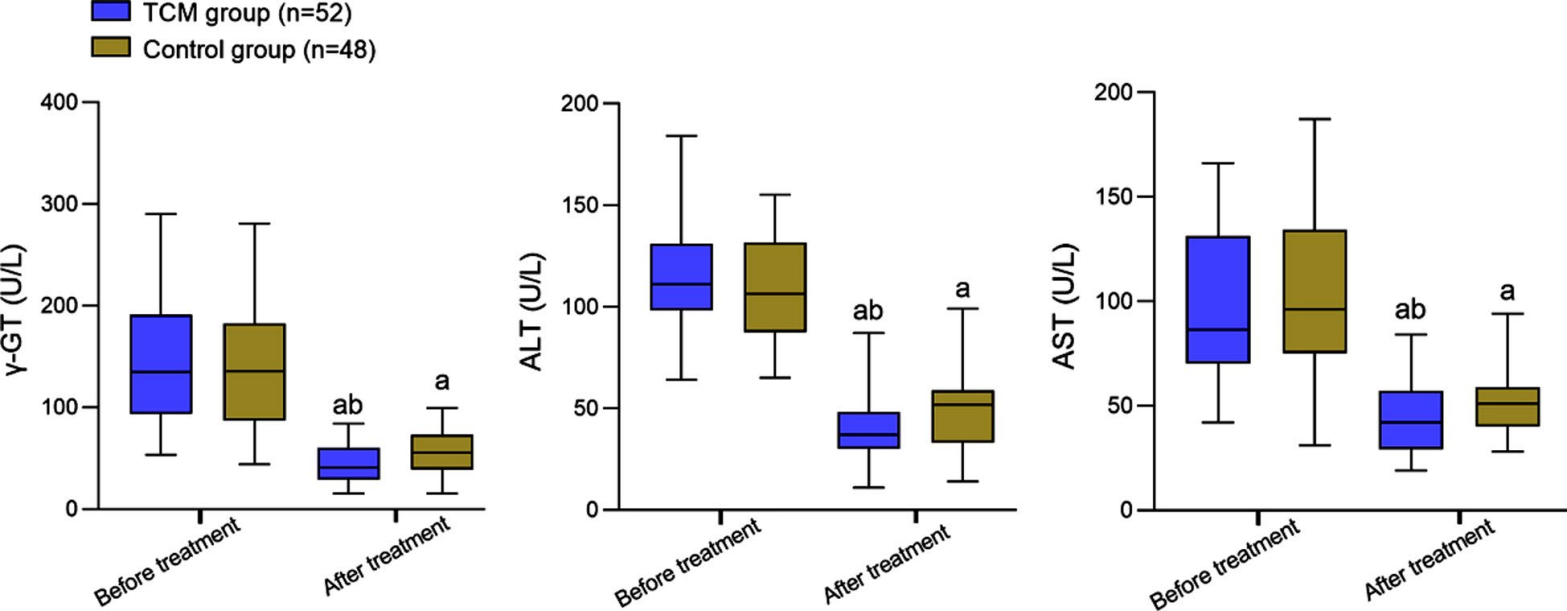
Comparison of liver function indicators between two groups; Compared with before treatment, ^a^*P*<0.05; Compared with the Control group, ^b^*P*<0.05; traditional Chinese medicine (TCM); Gamma-Glutamyl Transferase (γ-GT); aspartate aminotransferase (AST) or alanine aminotransferase (ALT).

Before the intervention, there was no significant difference in the levels of LDL-C, TG, and TC between the two groups (*P*>0.05). After the intervention, the levels of LDL-C, TG, and TC in both groups decreased and were significantly lower in the TCM group compared to the Control group (*P*<0.05) (Fig.1). Before the intervention, levels of γ-GT, ALT, and AST were comparable in the two groups (*P*>0.05). After the intervention, the levels of γ-GT, ALT, and AST in both groups decreased and were considerably lower in patients of the TCM group compared to the Control group (P<0.05) Fig.2. The overall effective rate in the TCM group (94.23%) was significantly higher than that in the Control group (79.17%) (P<0.05) (Table-III).

**Fig.3:**
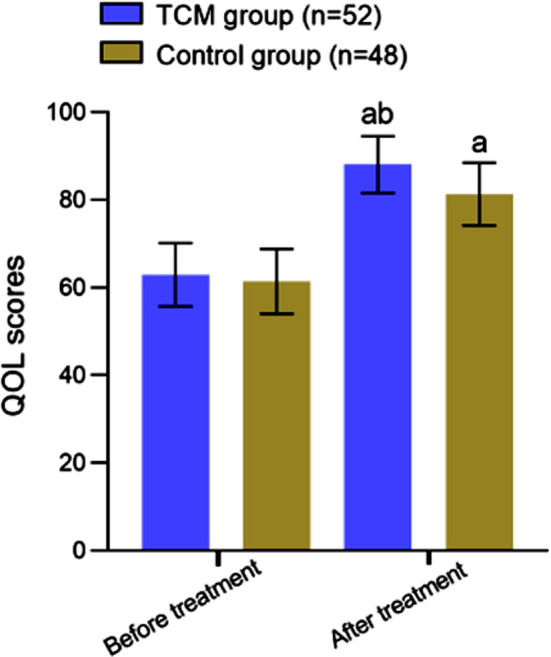
Comparison of quality of life between two groups; Compared with before treatment, ^a^*P*<0.05; compared with the Control group, ^b^*P*<0.05; Traditional Chinese medicine (TCM); Quality of life (QOL) .

Before the intervention, there was no significant difference in the QOL scores between the groups (*P*>0.05). While both methods of treatment increased the QOL scores, the QOL score of the TCM group was significantly higher than that of the Control group (P<0.05) Fig.3.

**Table-III:** Comparison of therapeutic effects of the two groups [n (%)].

Group	n	Significantly effective	Effective	Invalid	Overall effective
TCM group	52	31 (59.62)	18 (34.62)	3 (5.77)	49 (94.23)
Control group	48	22 (45.83)	16 (33.33)	10 (20.83)	38 (79.17)
*χ^2^*					5.008
*P*					0.025

## DISCUSSION

Our research results indicate that combining TCM sequential therapy with conventional measures can provide comprehensive intervention for NAFLD patients. Our study showed that the levels of blood lipids and liver function indicators in the TCM group were better than that in the Control group. The overall effective rate of TCM treatment (94.23%) was higher than that of the routine intervention (79.17%), and the QOL score of patients who received TCM treatment in addition to routine management was considerably higher than that of the Control group. It is plausible that compared to conventional interventions, TCM sequential therapy addresses the entire process of disease treatment and rehabilitation. It implements dialectical intervention based on the characteristics of the disease stage, focuses on adjusting intervention plans in a timely manner according to changes in the condition, and provides best intervention measures for patients. Previous studies have demonstrated that TCM sequential therapy implements comprehensive and targeted medical interventions according to a certain sequence and steps^11^-^14^ and is characterized by significant effectiveness, flexibility, and variability depending on the disease.^11^,^12^ This fully reflects the advantages of TCM in focusing on the whole organism.^11^,^12^,^18^ In recent years, the application value of TCM sequential therapy has been widely confirmed. Duan W et al.^19^ showed that TCM kidney tonifying sequential therapy can effectively improve the clinical symptoms, hormone levels, and ovarian function of patients with diminished ovarian reserve, enhance ovulation quality and pregnancy rate, and improve quality of life. A multicenter randomized controlled study confirmed that TCM sequential therapy has significant advantages in improving pregnancy outcomes in patients with endometriosis-related infertility.^10^

Our study provides evidence of the intervention value of TCM sequential method in treating NAFLD. TCM classifies NAFLD into categories such as “dampness obstruction”, “bloating”, “liver stagnation”, and “phlegm syndrome”,^18^,^20^ and suggests that NAFLD is mainly caused by factors such as overeating, loss of emotional flow, the sensation of dampness and heat, and prolonged illness leading to liver dysfunction, loss of spleen function, cessation of water and dampness, endogenous phlegm turbidity, and qi stagnation and blood stasis.^20^,^21^ The lesion is located in the liver and involves the spleen, stomach, gallbladder, and kidneys. The condition is relatively complex and, therefore, cannot be effectively treated with a single medication. During the treatment, diet, exercise, and medication should be combined, and a comprehensive TCM treatment plan should be adopted, with medication adjusted according to the patient’s condition.^19^-^21^

The formula used in our study focuses on the core treatment methods of soothing the liver and strengthening the spleen, drying dampness, and resolving phlegm. The diagnosis and treatment approach takes the disease site as the starting point, combines the relationship between organs, and infers treatment methods based on the pathogenesis, aiming to consider both the symptoms and the body. Sun T et al.^22^ used polyene phosphatidylcholine capsules, and showed that they can significantly improve liver function and blood lipid levels. Zhou Z et al.^23^ also showed that herbs such as licorice, coix seed, and Asteraceae, as well as active Chinese herbal ingredients, can clear dampness and heat. This is consistent with the conclusion of our study. In addition, aerobic exercise has been shown to promote fat breakdown in various tissues, enhance oxidase activity, activate gamma receptors, and alter levels of adipokines, thereby helping to improve NAFLD.^24^ The study by Zhang HJ et al.^25^ evaluated the effects of different intensities of aerobic exercise on NAFLD patients at six and 12 months and showed that patients exhibited enhanced liver adaptability to exercise and reduced liver fat content.

In our study, in addition to regular aerobic exercise, patients in the TCM group were also guided to engage in traditional Chinese martial arts exercises such as Tai Chi, Baduanjin, and Yijinjing. There are studies and indications that Tai Chi can significantly reduce waist circumference, blood sugar levels, and insulin resistance in obese adults, and increase high-density lipoprotein cholesterol levels.^26^,^27^ TCM sequential therapy in our study also included acupoint therapy. The potential mechanism by which acupoint stimulation may affect NAFLD is still unclear. However, there are some indications that acupoint stimulation may improve the main pathological manifestations of NAFLD, including insulin resistance, chronic inflammation, and oxidative stress.^28^ Studies have also shown that acupoint therapy is more effective in relieving liver damage or regulating blood lipid levels and has a lower incidence of adverse events.^28^,^29^ In addition, there are more than one hundred types of possible TCM therapies, including acupuncture and moxibustion, massage and massage, acupoint catgut embedding, cupping, ointment, and ear point therapy. These approaches have the advantages of unique therapeutic effects, rapid action, long history, and simple and cost-effective testing.^20^ Related studies have also shown that TCM therapies can effectively treat NAFLD, especially in terms of affecting liver fat, lipid metabolism, and insulin resistance.^30^,^31^

The results of our study further confirmed previous reports that TCM sequential therapy can enhance the overall physical function of NAFLD patients, reduce the side effects of traditional drugs, and improve the overall treatment effect.^22^,^23^ TCM sequential method focuses on overall balance and regulation, using a combination of herbs, exercise guidance, and acupoint stimulation to treat NAFLD, which is shown to be a promising clinical treatment for patients with NAFLD.^25^–^27^ Therefore, our study can provide new ideas and practical references for the health management of NAFLD.

### Limitations:

This is a small-scale single-center retrospective analysis with the short follow-up time. Therefore, our results do not assess the long-term prognosis of NAFLD patients treated with TCM sequential therapy. Additionally, TCM sequential therapy has certain limitations, such as unknown pharmacological effects and lack of clinical experimental evidence. Due to the unique use of herbs, which often involves a combination of multiple herbs, it is often challenging to find specific and effective ingredients. Finally, in the design of TCM therapy, the main considerations include the selection of Chinese medicine, dosage, administration route, and administration plan. Although TCM is usually associated with fewer side effects and is suitable for long-term use, its efficacy and mechanism need to be further validated through rigorous clinical research, and treatment may vary from person to person.

## CONCLUSION

Combining TCM sequential therapy with routine intervention can effectively regulate blood lipid levels, restore liver function, and improve the effectiveness of disease treatment and the quality of life of NAFLD patients.

### Author’s contributions:

**LZ:** Study design, Concept, literature search, manuscript writing and manuscript revision and validation and is responsible for the integrity of the study.

**YQ**, **HW** and **HX:** Data collection, analysis, interpretation and critical review.

All authors have read and approved the final manuscript.
